# *In vitro* growth competition experiments that suggest consequences of the substandard artemisinin epidemic that may be accelerating drug resistance in *P*. *falciparum* malaria

**DOI:** 10.1371/journal.pone.0248057

**Published:** 2021-03-09

**Authors:** Matthew R. Hassett, Paul D. Roepe

**Affiliations:** Dept. of Chemistry and Dept. of Biochemistry & Cellular & Molecular Biology, Georgetown University (MRH, PDR), Washington, DC, United States of America; Institut national de la santé et de la recherche médicale - Institut Cochin, FRANCE

## Abstract

Over the past decade, artemisinin (ART)-combination therapies (ACTs) have shown declining efficacy within Southeast Asia (SEA). These resistance-like phenomena manifest as a delayed clearance phenotype (DCP) in some patients treated with ACTs. ACTs are currently the recommended treatment for *P*. *falciparum* infections by the World Health Organization (WHO), and they are our last line of defense to effectively treat all strains of malaria. Acceleration of antimicrobial resistance (AMR) is often theorized to be exacerbated by the use of subtherapeutic dosages of drugs (“substandard” drug), which for ACTs has been well documented over the last decade. Troublingly, in 2017, the WHO estimated that nearly 1 in 10 medical products tested in low- and middle-income countries failed to meet quality standards. We have developed a tissue culture-based approach for testing possible connections between substandard treatment and the spread of ACT resistant blood stage forms of *P*. *falciparum*. Via sequencing of *pfk13*, a molecular marker that is predictive for ART resistance (ARTR), we monitor competition of sensitive vs resistant strains over time and under various conditions and define conditions that favor emergence of ARTR parasites. Our findings help to define the conditions under which substandard drug treatments might favor the proliferation of mutant PfK13-mediated drug resistant strains over drug sensitive.

## Introduction

Artemisinin (ART)-based Combination Therapies (ACTs) are currently the frontline treatments for uncomplicated *Plasmodium falciparum* malaria infections recommended by the World Health Organization (WHO) and are our last line of defense to effectively treat all strains of malaria. ACTs are comprised of an ART-based drug, which has a short half-life but can reduce parasite burden by orders of magnitude within hours, and a longer lasting partner drug that prevents recrudescence. Treatment regimens outlined by the WHO typically recommend three days of ACT administration with doses separated by 24 h (the exception to this being artemether/lumefantrine which is given twice a day, separated by 8 h, for 3 days).

Despite early success in reducing the worldwide burden of malaria, a harbinger of ART resistance (ARTR) was identified in western Cambodia in 2006–2007 when increased parasite clearance times were identified in patients after artesunate monotherapy [1]. Parasites exhibiting this delayed clearance phenotype (DCP) have been spreading from the initial epicenter in western Cambodia throughout the Greater Mekong Subregion (GMS) [2]. A number of studies have identified the emergence of other founder populations throughout the GMS in the years following the identification of DCP in western Cambodia [3–9]. However, currently, only five countries have identified ACTs that are “failing”; Cambodia, Thailand, Myanmar, Vietnam, and Laos. The WHO characterizes ACTs as failing if the proportion of patients that are parasitaemic on day 3 is above 10%, or the proportion of treatment failure by day 28 or 42 is above 10% after ACT treatment [10]. Isolated ACT treatment failure has also been identified in India and on the African continent, but the number of documented cases remains small and the WHO has not classified any ACTs as failing in these areas [11–17].

In 2014, non-synonymous single nucleotide polymorphisms (SNPs) were identified on a molecular marker that correlated with DCP for GMS isolates [18]. This single exon gene on chr 13 was named *pfk13* and encodes a 726 amino acid protein that has three distinct domains, including a Kelch-like propeller domain. A number of different amino acid substitutions in the propeller domain including Y493H, R539T, I543T, and C580Y have since been associated with ARTR using the “ring-stage survival” assay (RSA), which examines the outgrowth of early ring stage parasites after bolus dose with an ART-based drug at a pharmacologically relevant concentration [19]. A year later, reverse genetic experiments confirmed the association between specific *pfk13* SNPs and RSA-quantified ARTR [20]. The degree of ARTR appears to depend on both the specific *pfk13* mutation as well as the genetic background of a strain, which suggests that additional genetic factors likely regulate DCP [20]. Any link between *pfk13* SNPs and DCP has yet to be established in Africa [11–15, 21].

It is critical to understand how resistance ACTs (to either one or both drugs) has developed and spread despite the remarkable success of ACTs in reducing malaria related deaths between 2000–2015 [22]. It is currently unclear to what degree different factors are catalyzing the spread of ACT resistances within the GMS, however, currently, the only areas harboring high frequencies of DCP parasites remain confined to the GMS. Spread of strains harboring *pfk13* mutations to Africa would be devastating, as the majority of deaths due to *P*. *falciparum* infections occur in Africa. As stated, one unique feature of ART-based drugs when compared to other antimalarials is a very short biological half-life. Without an effective ACT partner drug, re-emergence of infection is possible, since ART monotherapy has long been known to be less effective than combination therapy and often results in recrudescence [23]. An insufficient or subtherapeutic dose of the ART-based drug in the ACT places an even heavier burden on the ACT partner drug. Therefore, lack of proper adherence to WHO recommended ACT regimens in some circumstances may be one factor driving accelerated emergence of DCP.

Before the emergence of malarial parasites showing DCP, nearly 78% of patients surveyed in Cambodia were receiving ART monotherapy [24], leading to conditions that promoted recrudescence of drug tolerant parasites and the evolution of mutant *pfk13*-mediated ARTR [2]. In addition, in the fall of 2017, the WHO estimated that nearly 1 in 10 medical products in low- and middle-income countries were substandard or falsified, including 11.8% of all antimalarials tested [25]. Survey of substandard and counterfeit medicines from around the world shows that substandard or falsified medicines are not isolated to a specific region [26–40]. Results from several laboratories have suggested that fitness costs associated with drug resistance phenomena can change with environmental conditions (e.g. the presence of variable [drug]) [41–45] which implies that use of substandard antimalarial medications may affect the spread of ARTR malarial parasites.

That is, fitness costs are a well-known byproduct of drug resistance conferring mutations. Previous work has shown that amino acid substitutions in the *P*. *falciparum* chloroquine resistance transporter (PfCRT) that confer resistance to amino quinoline-based antimalarial drugs can also impact parasite fitness [46, 47]. In this study, we use a well characterized tissue culture model for PfK13-mediated ARTR comprised of isogenic strains “CamWT” and “CamWT^C580Y^” [20] to examine whether *pfk13* mutation associated with ARTR alters intraerythrocytic parasite fitness under different conditions. In this model, differences in parasite genetic background are controlled, so any observed change in parasite fitness is solely related to *pfk13* mutations. Conveniently, because the half-life of ART-based drugs is short, correlation between dose and plasma concentration is linear and predictable [48–52]. We therefore modelled the effects of varied subtherapeutic dosing as well as substandard schedule of drug administration on the possible spread of ARTR malarial parasites.

## Materials and methods

### Materials

Parasite culture plastics were from Fisher (Hampton, NH). All chemicals were reagent grade or better, purchased from commercial sources, and used without further purification. RBCs for parasite culture were from Valley Biomedical (Winchester, VA, catalog number HB1055).

### Methods

#### Cell culture

*P*. *falciparum* strains CamWT (ART-sensitive; ARTS) and CamWT^C580Y^ (ART-resistant; ARTR), kindly provided by Professor David A. Fidock (Columbia University) [20], were maintained essentially as described previously [53] with minor modifications. In brief, cultures were maintained in a custom blend gas mix (5% CO_2_/ 5% O_2_) at 2% hematocrit in Complete Media [RPMI 1640 supplemented with 0.5% Albumax II, 25 mM HEPES (pH 7.4), 24 mM NaHCO_3_, 11 mM glucose, 0.75 mM hypoxanthine, and 20 μg/L gentamycin]. Parasitemia was monitored and recorded via Giemsa staining and adjusted by adding fresh RBCs and fresh complete media. Before being added to cultures, RBCs were washed with incomplete medium [RPMI 1640, 24 mM NaHCO_3_, 11 mM glucose, and 0.75 mM hypoxanthine (pH 7.4)].

#### Competition experiments

**Fig 1:**
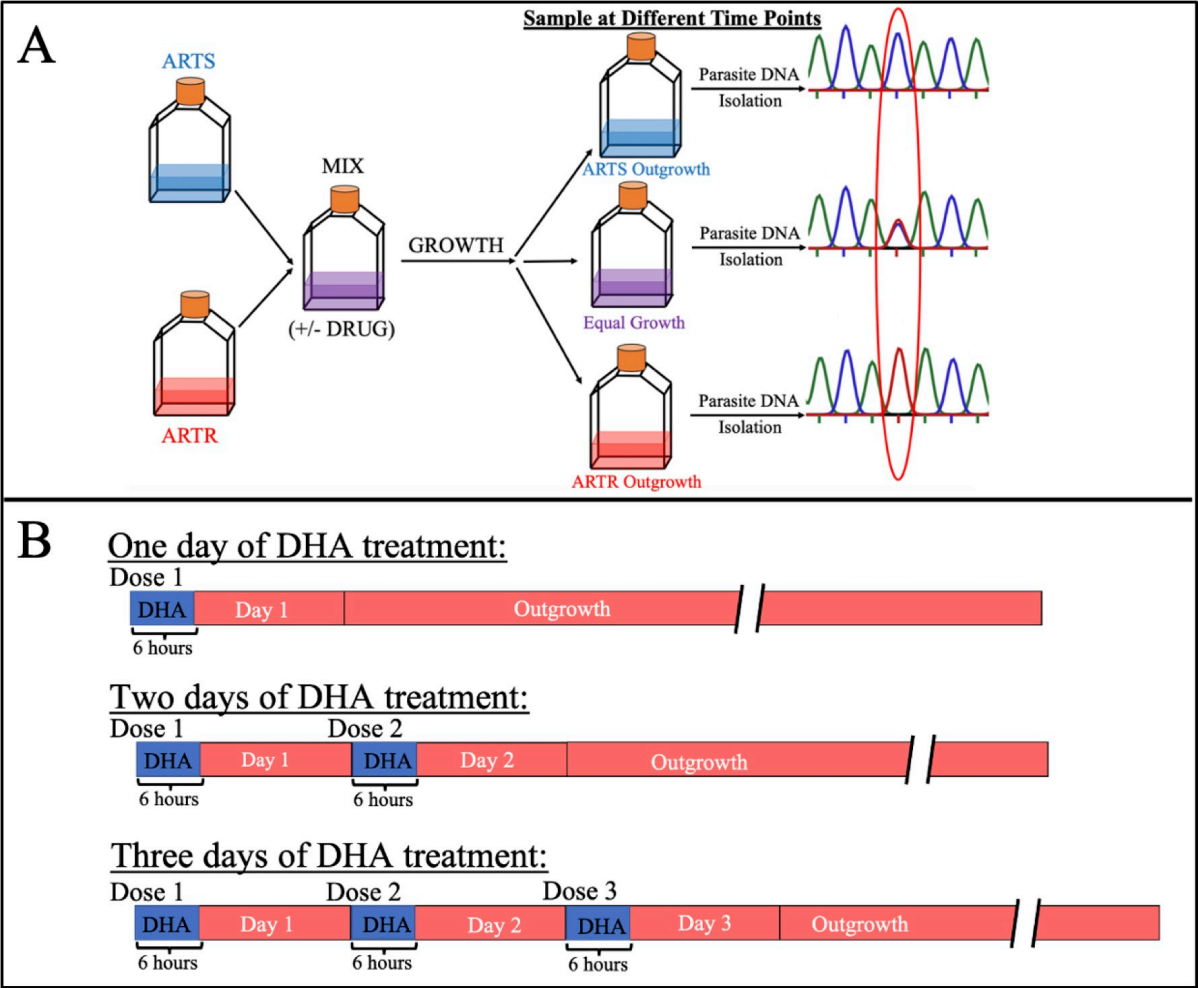
Schematic of growth competition experiments. (A) ARTS and ARTR lines are combined at defined ratios in either drug free media, or media containing dihydroartemisinin (DHA), the active metabolite of all ART-based drugs used in the clinic. Mixed cultures that are exposed to drug are bolus dosed for 6 h, after which they are washed three times with complete media. Outgrowth of mixed cultures in complete media is monitored by amplification of *pfk13* and sequencing the PCR product to determine the ratio of ARTS to ARTR (e.g. the ratio of C580 vs Y580 codon abundance). (B) Mixed cultures are dosed with drug to mimic WHO-defined schedules of ACT-administration or substandard treatment by titrating DHA 6h bolus dose and/or altering the dosing schedule (e.g. < or = 3).

Laboratory strains CamWT and CamWT^C580Y^ were mixed at defined ratios in either drug free media or media containing various concentrations of dihydroartemisinin (DHA), the active metabolite of all ART-based drugs used in the clinic. Mixed cultures were asynchronous and initially seeded at 3% parasitemia. For competition in the presence of drug, mixed cultures were bolus dosed for 6 h and then washed three times with complete media. Subsequent 6 h bolus doses were performed 24 h after the preceding dose and washed three times with complete media (Fig 1B). Outgrowth was monitored visually by Giemsa smears with media changes at least every 3^rd^ day for cultures with submicroscopic parasitemia. To monitor competition of the mixed cultures, aliquots were taken for *pfk13* DNA sequencing and relative outgrowth of the strains quantified vs time. Total number of parasites indicated in Figs 4–6 corresponds to total growth of parasites (the total that would be obtained without routine culture dilutions, see above).

#### Modelling parasite competition

To further model growth competition between CamWT vs CamWT^C580Y^, growth rates were first measured for monocultures of each strain in triplicate. Theoretical growth competition for a mixed culture was then calculated using growth rates obtained for each strain alone and the starting number of parasites using Eq 1:

$$Ratio\;CamWT\;parasites=\frac{X}{Y}e^{0.452\times t}$$ (1)

where X is the number of CamWT parasites, Y is the number of CamWT^C580Y^ parasites and t is the number of replication cycles.

#### DNA extraction and *pfk13* amplification

Genomic DNA from iRBC pellet was isolated using the DNeasy Kit from Qiagen (Valencia, CA) and *pfk13* fragments were amplified from 100 ng of purified genomic DNA isolated from mixed cultures using the Taq PCR Master Mix Kit (Qiagen, Valencia, CA). Briefly, *pfk13* was amplified using 200 nM of forward and reverse primers (Forward primer 5’-3’: GCAGCAAATCTTATAAATGATGATTCTGG; Reverse primer 5’-3’: CGGAGTGACCAAATCTGGGAAC). Amplification conditions were as follows: 94 ^o^C for 3 minutes; 35 cycles of 94 ^o^C for 30 seconds, 55 ^o^C for 30 seconds, and 60 ^o^C for 1min/kb of DNA; followed by 60 ^o^C for 10 minutes. Amplified PCR samples (1.5 kbp) were purified using the QIAquick Gel Extraction Kit (Qiagen, Valencia, CA). Samples were sequenced to identify the ratio between CamWT and CamWT^C580Y^ (identified by the codon 580 sequence) at each experimental timepoint via Sanger sequencing and measuring the area under the curve for each signal corresponding to the two different possible bases at this position.

## Results

### Quantification of competitive growth

Quantification of growth competition for mixed malarial parasite cultures has been previously described; typically, unique genetic markers or a reporter line expressing a fluorescent marker are used to differentiate between two strains as they grow in mixed culture over time [46, 47]. In this study, we used isogenic parasite lines that only differ at a single *pfk13* codon to test the potential impact that ARTR-associated mutant PfK13 protein has on the fitness of malarial parasite iRBC stages in the presence of different dosages of dihydroartemisinin (DHA).

Fig 1 details our tissue-culture based approach (A) and various culture dosing schedules (B). Briefly, ARTS and ARTR parasites are combined in culture in the presence or absence of drug (Fig 1A). In the presence of drug, the mixed culture is bolus dosed for 6 h with DHA, the active metabolite of all ART-based drugs (B). The concentration of drug varied from 700 nM (~ 100% of the pharmacologically relevant dose) to 43.8 nM (~ 6.25% of dose used in the clinic). Growth of mixed cultures vs time was monitored by aliquoting the growing culture, purifying genomic DNA, amplifying a 580 codon containing region of *pfk13* (see Methods), and sequencing to determine the ratio of ARTS (strain CamWT) to ARTR (strain CamWT^C580Y^) parasites in the culture. (Fig 1A).

### Fitness of ARTS vs ARTR parasite strains

The fitness of a few ARTR lines has been examined in previous work [41–45], however only one of these [42] examined competitive fitness in the presence vs absence of drug. In this work Hott *et al*. [42] examined a laboratory selected strain of *P*. *falciparum* that did not express PfK13 protein harboring a propeller domain mutation validated for conferring ARTR in and of itself, but the strain did contain *pfk13* encoding an E208K amino acid substitution that appeared to be unnecessary for conferring ARTR. No prior work to our knowledge has examined the effects of varying drug concentration and none have compared single vs multiple drug bolus dosing to mimic changing WHO recommended treatment schedules.

**Fig 2:**
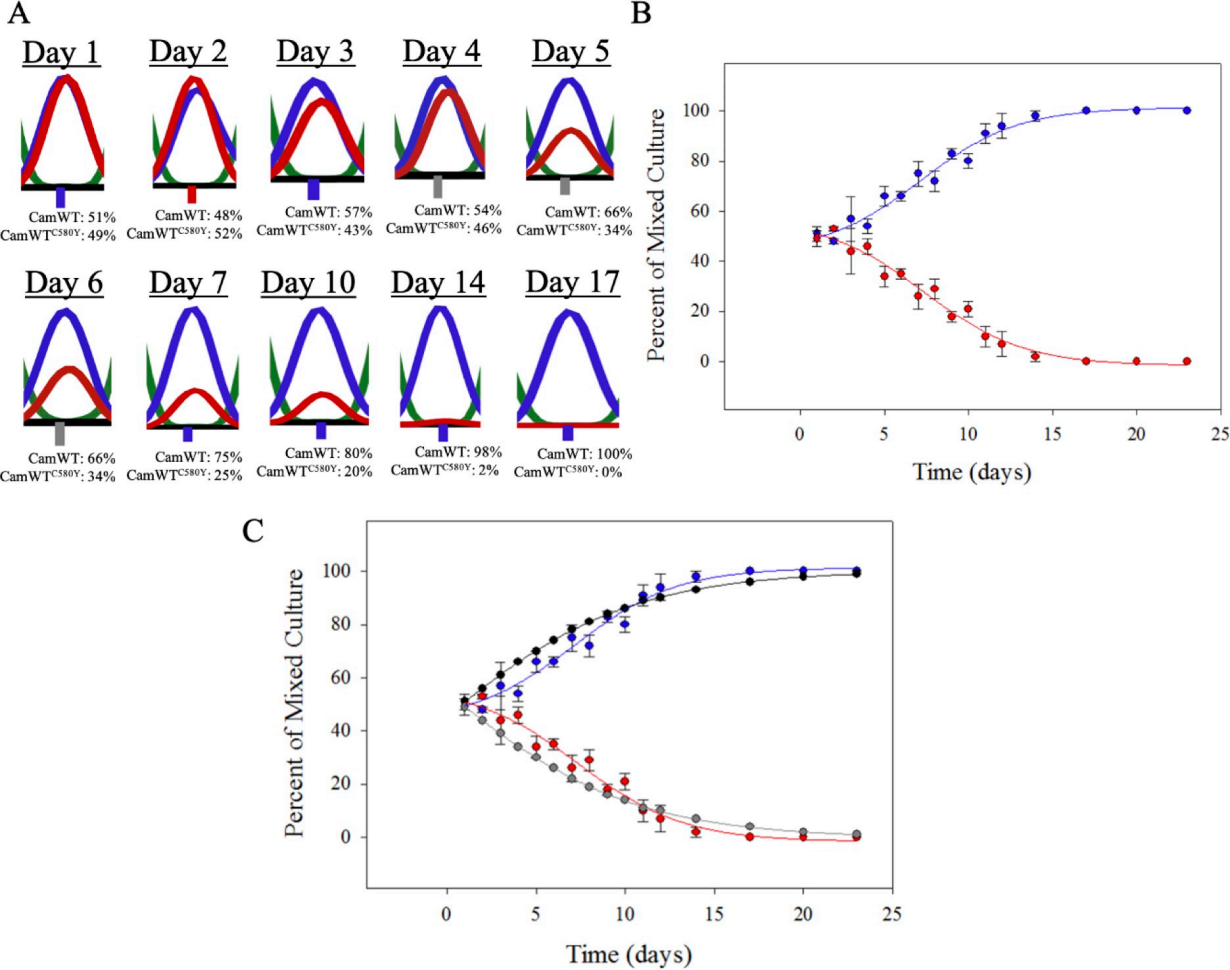
Growth competition with no drug. (A) CamWT and CamWT^C580Y^ were mixed at a 50:50 ratio in complete media without drug. Within 7 days, the ARTS strain (CamWT, blue), has outcompeted the ARTR strain (CamWT^C580Y^, red). By Day 17, the mixed culture is virtually 100% ARTS. This suggests that there is a fitness cost associated with expression of mutant PfK13 protein within CamWT^C580Y^ and that CamWT is more fit. (B) Data from sequencing chromatograms are plotted as a function of time and curve fitted. These control data indicate that if there is an environment that favors the outgrowth of the resistant strain, it is not due to an intrinsic fitness advantage. Data shown are the average of 3 independent growth trials, +/- S.E.M. C) Modeling mixed culture outgrowth. Monocultures of CamWT and CamWT^C580Y^ were grown in triplicate to assess growth rate for each strain. Using monoculture growth rates, growth of mixed cultures was modeled and compared to experimentally obtained data from mixed cultures. Theoretical (calculated, see Eq 1) growth of CamWT (black symbols) and CamWT^C580Y^ (gray) in a 50:50 mixed culture are very similar to experimentally observed results for CamWT (blue symbols) and CamWT^C580Y^ (red) mixed at the same ratio as shown in Fig 2B.

C580Y/PfK13 is the most common PfK13 propeller domain amino acid substitution linked to ARTR throughout SEA [5, 54–56]. We first examined the relative intrinsic fitness of CamWT and CamWT^C580Y^ parasites in the absence of drug. We find that in the absence of drug, CamWT red blood cell growth is faster than that of CamWT^C580Y^ (Fig 2), indeed, mixed culture seeded at a 1:1 (“50:50”) ratio becomes predominantly CamWT in ~ 2 weeks. In Fig 2A, a sampling of sequencing chromatograms depicts initial seeding conditions as well as the outgrowth of CamWT (blue peak) vs the disappearance of CamWT^C580Y^ (red peak). The outgrowth of CamWT was then plotted and fitted as shown in Fig 2B. These data are critical for interpretation of subsequent competition experiments with mixed cultures +/- various [drug]. That is, in the absence of drug, CamWT (the genetically matched ARTS strain) is more fit, which indicates that if any condition is found where the ARTR strain outcompetes the ARTS, this is not due to any intrinsic growth advantage, instead, the environment that results in ARTR strain outgrowth must confer an ARTR growth advantage.

We also quantified growth of CamWT and CamWT^C580Y^ monocultures in the absence of drug and used these growth rates to model competitive outgrowth of mixed cultures. We find that growth rates for the two strains (CamWT and CamWT^C580Y^) were significantly different. This is in contrast to some earlier results [41], but consistent with other data that found a significant fitness cost due to PfK13 C580Y substitution [43]. Computed competitive outgrowth using these monoculture growth rates showed excellent correlation with experimentally measured competitive growth (Fig 2C).

**Fig 3:**
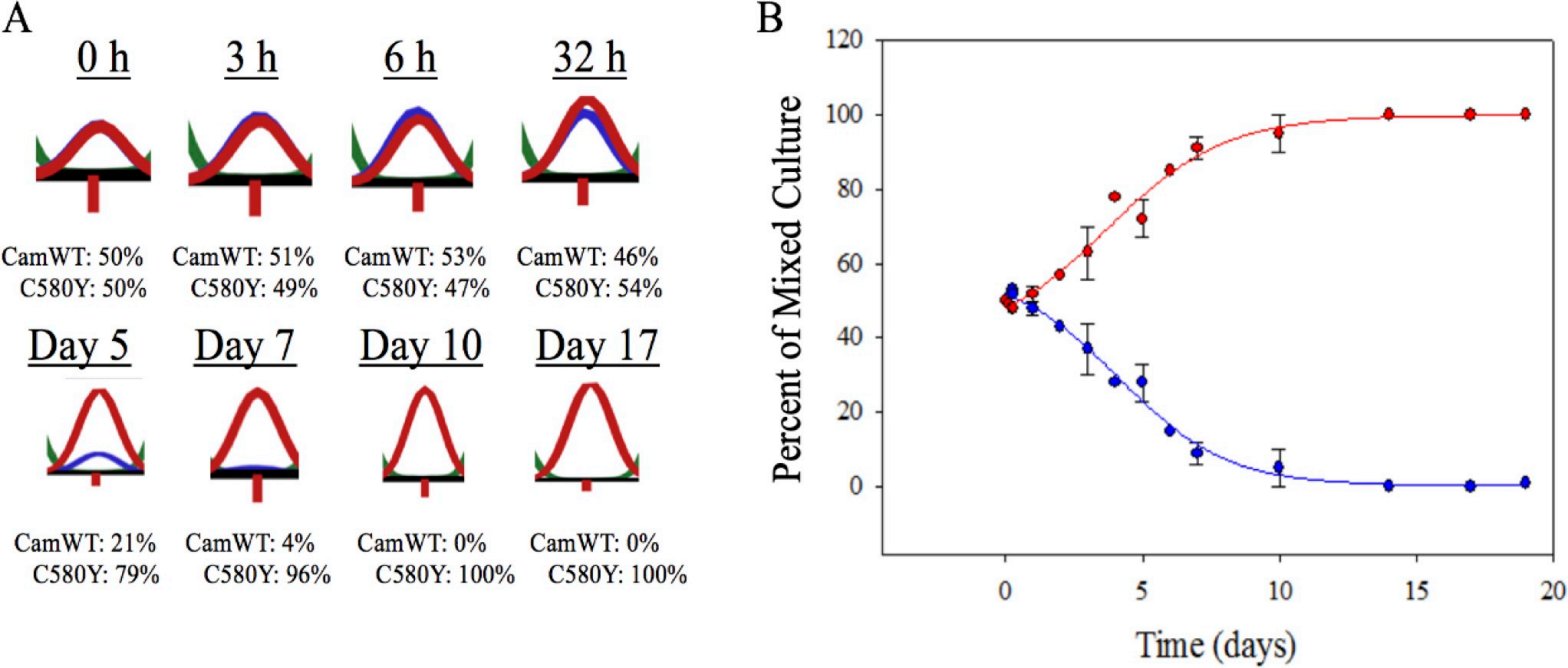
Mixed growth competition in drug. (A) CamWT and CamWT^C580Y^ were mixed at a 50:50 ratio in complete media and bolus dosed for 6 hr with 700 nM DHA. Contrary to growth competition in no drug (Fig 2), outgrowth of CamWT^C580Y^ (red) is seen relative to CamWT (blue). By day 7, CamWT^C580Y^ has outcompeted CamWT, and by Day 10 the mixed culture is essentially 100% CamWT^C580Y^. (B) Data from sequencing chromatograms are plotted as a function of time and curve fitted. Error bars represent the SEM of at least three independent trials.

In order to quantify growth competition in the presence of drug, the two strains were mixed at a 50:50 ratio and bolus dosed with DHA (see Methods). Within one week, a single 700 nM dose of DHA exerted sufficient selective pressure to facilitate the outgrowth of CamWT^C580Y^ over CamWT (Fig 3). No immediate change in the ratio between CamWT and CamWT^C580Y^ was seen in the first 32 h after drug treatment (Fig 3A), despite rapid DHA induced killing of all parasites (not shown, see below). Once measurable outgrowth began, CamWT^C580Y^ outcompeted CamWT (Fig 3A and 3B). In contrast to Fig 2, which shows that CamWT is more fit in the absence of drug, we observe proliferation of CamWT^C580Y^ in the presence of a single therapeutic dose of DHA.

#### Substandard drug treatment

**Fig 4:**
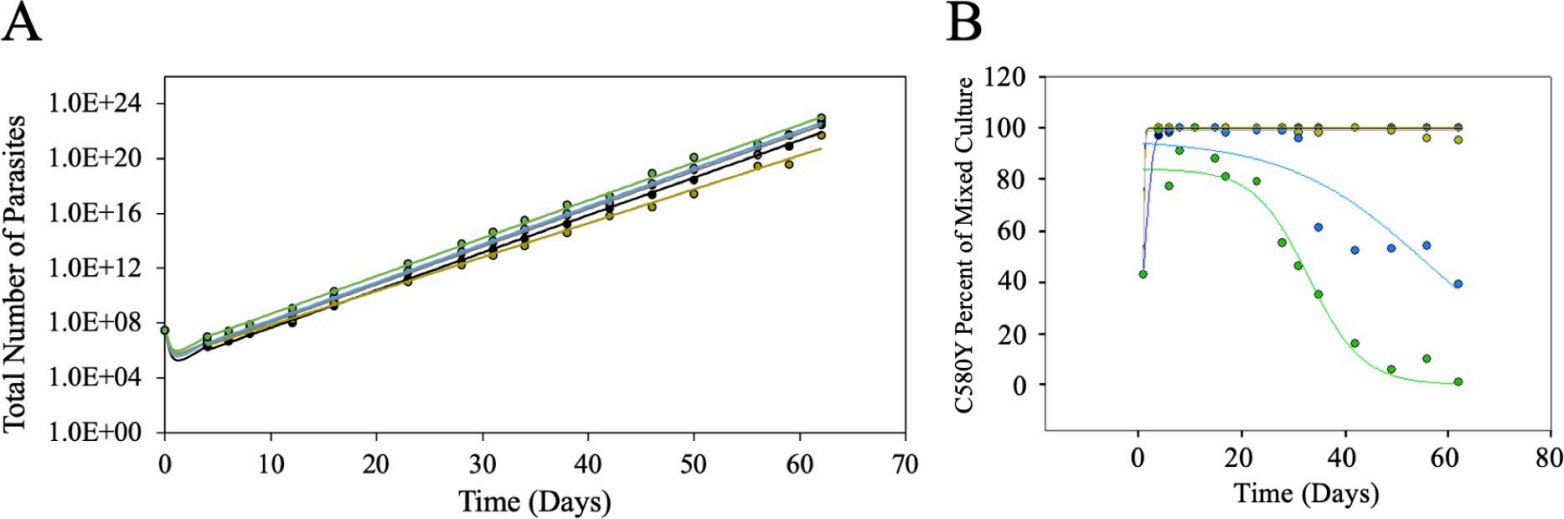
Mixed culture outgrowth following a single DHA bolus dose. CamWT and CamWT^C580Y^ cultures were mixed at a 50:50 ratio in complete media and bolus dosed with DHA at various concentrations (700 nM = black; 350 nM = gray; 175 nM = yellow; 87.5 nM = blue; 43.8 nM = green). (A) Total parasite outgrowth is plotted vs time. Growth was determined by Giemsa smears. Earlier timepoints (e.g. < day 4) those before visible growth via Giemsa smears could be identified were extrapolated from exponential growth rates once visible growth had resumed. (B) Percentage of the culture that was CamWT^C580Y^ was plotted as a function of time for the various DHA bolus doses (700 nM = black; 350 nM = gray; 175 nM = yellow; 87.5 nM = blue; 43.8 nM = green). DHA provides a fitness advantage for CamWT^C580Y^ lines over CamWT lines, however, at lower concentrations of drug the advantage is not substantial enough to deter the re-emergence of CamWT after ~ 3 weeks. Higher concentrations of drug provide a sustained fitness advantage vs CamWT for at least 9 weeks.

We next tested what impact substandard concentrations of drug (meaning < 100% therapeutic = 700 nM DHA since this is near plasma [DHA] for large numbers of patients [48–52]) or substandard adherence (modelled as < 3 bolus doses) would have on the relative fitness of ARTS vs ARTR parasites. Initially, we examined a single bolus dose at varied concentration to model substandard drug dose. CamWT and CamWT^C580Y^ were mixed at 50:50 ratio and bolus dosed with variable drug (700 nM, 350 nM, 175 nM, 87.5 nM, and 43.8 nM) representing 100%, 50%, 25%, 12.5%, and 6.25% of a pharmacologically relevant dose. Total parasite growth was plotted as shown in Fig 4A. Earlier timepoints (those before visible growth via Giemsa smears could be identified) were extrapolated from exponential growth rates once visible growth had resumed. An immediate ~ 1–2 log drop in parasitemia was computed for mixed cultures after single bolus dose drug treatment with any increased magnitude of the drop depending on [DHA] (Fig 4A) or number of bolus doses (below). Once cultures recovered from drug treatment, growth rates for all mixed cultures were similar (Fig 4A). As in Fig 3, we find that all concentrations of drug examined initially provided a growth advantage for the CamWT^C580Y^ line (Fig 4B), however, interestingly, at lower concentrations (12.5% and 6.25%, blue and green symbols, respectively) we observe dose dependent outgrowth of CamWT after ~ 3 weeks (Fig 4B). This suggests that at lower doses, which provide an initial fitness advantage for the ARTR strain, there is not sufficient selective pressure to provide a long-term growth advantage to ARTR parasites.

**Fig 5:**
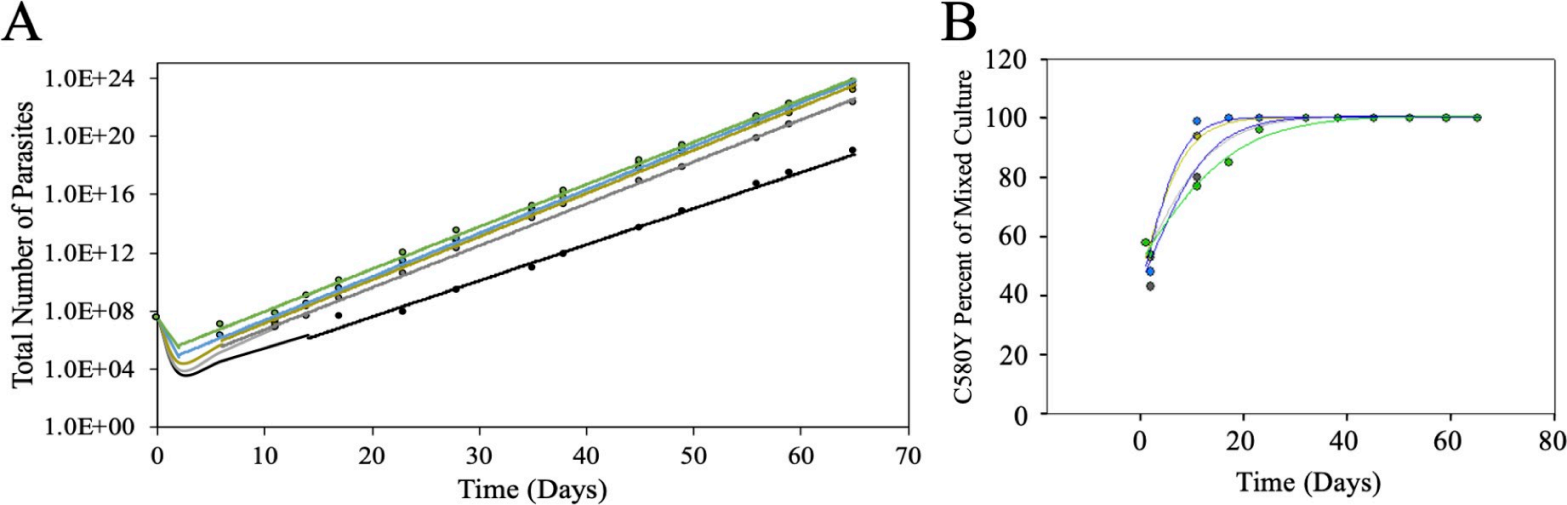
Mixed culture outgrowth following 2 X DHA bolus dosing. CamWT and CamWT^C580Y^ cultures were mixed at a 50:50 ratio in complete media and dosed with DHA at various concentrations (700 nM = black; 350 nM = gray; 175 nM = yellow; 87.5 nM = blue; 43.8 nM = green). Mixed cultures are bolus dosed for 6 h, after which they are washed three times with complete media. Mixed cultures are bolus dosed again, 24 h after the first dose (to mimic the time between doses in the clinic) and washed again three times with complete media. (A) Total parasite outgrowth is plotted as a function of time. Visible growth was determined by Giemsa smears and earlier timepoints were extrapolated from growth rates after visible growth was measured. (B) Percentage of the culture that was CamWT^C580Y^ was plotted as a function of time for the various DHA bolus doses.

Next, we examined outgrowth after two consecutive days of DHA bolus dosing (substandard adherence). Mixed cultures were bolus dosed a second time (at the same concentration as the first dose), 24 h after the first dose. Larger drops in parasitemia, ~ 3–4 log, were calculated after two bolus doses, with the magnitude of the drop again depending upon [DHA] (Fig 5A). As with the single dose, once visible growth via Giemsa smears is resumed growth rates for all mixed cultures were similar. After two bolus doses, the initial outgrowth of CamWT^C580Y^ vs CamWT is delayed when compared to the single dose (Fig 4B). However, unlike the behavior upon single dose, two bolus doses, regardless the [DHA] provided a clear fitness advantage for CamWT^C580Y^ parasites that persisted for at least 9 weeks.

**Fig 6:**
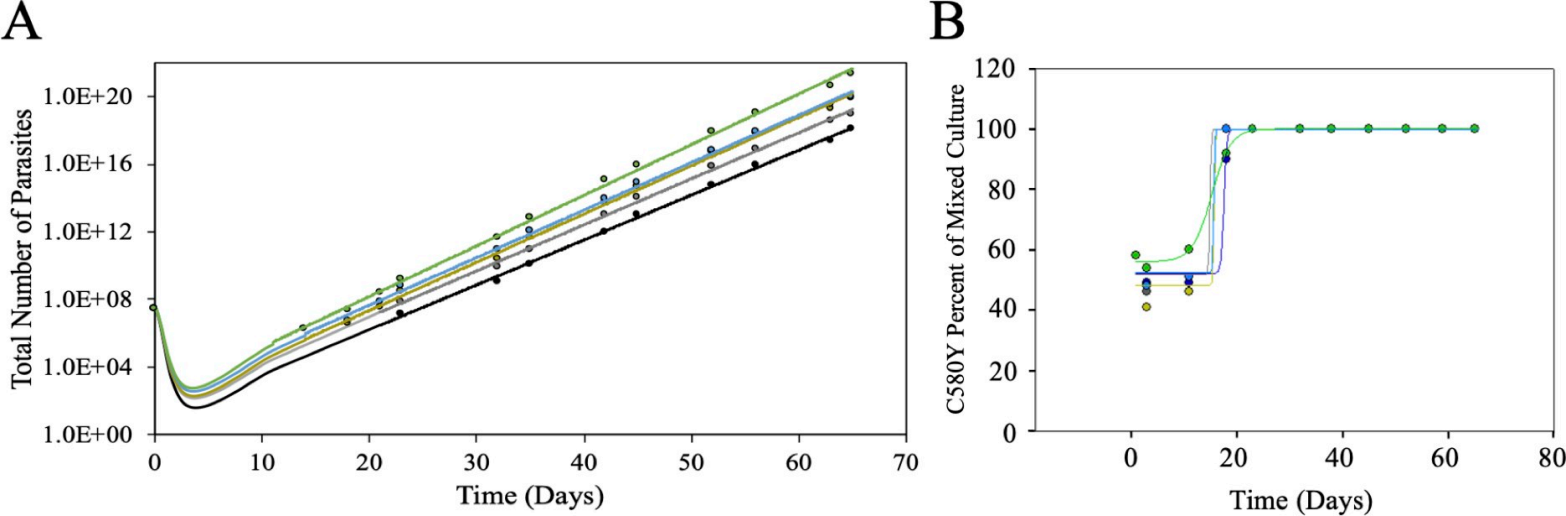
Mixed culture outgrowth following 3 X DHA bolus dosing. CamWT and CamWT^C580Y^ cultures were mixed at a 50:50 ratio in complete media and dosed with DHA at various concentrations (700 nM = black; 350 nM = gray; 175 nM = yellow; 87.5 nM = blue; 43.8 nM = green). Mixed cultures are bolus dosed for 6 h, after which they are washed three times with complete media. Mixed cultures are bolus dosed again, 24 h after the first dose (to mimic the time between doses in the clinic) and washed three times with complete media. A third bolus dose is administered 24 h after the second, and again, mixed cultures are washed three times with complete media. (A) Total parasite outgrowth is plotted as a function of time. Visible growth was determined by Giemsa smears and earlier timepoints were extrapolated from growth rates after visible growth was measured. (B) Percentage of the culture that was CamWT^C580Y^ was plotted as a function of time for the various DHA bolus doses.

Three consecutive days of DHA bolus treatment would most closely model full adherence within the clinic (Fig 6). Not surprisingly, a larger initial parasite “clearance” was calculated for the cultures following three bolus doses. Extrapolation of parasitemia estimates ~ 4–5 log reduction in initial parasitemia following three doses (Fig 6A), and again, once visible growth has been re-established, all mixed cultures grew at similar rate for the remainder of the experiment. An even longer delay in the outgrowth of CamWT^C580Y^ vs CamWT was seen after 3 doses (Fig 6B). There is essentially no change in the ratio between CamWT and CamWT^C580Y^ until day 18, at which point a much more rapid jump in the ratio from 50% to near 100% CamWT^C580Y^ was observed relative to single and twice bolus exposure experiments.

**Fig 7:**
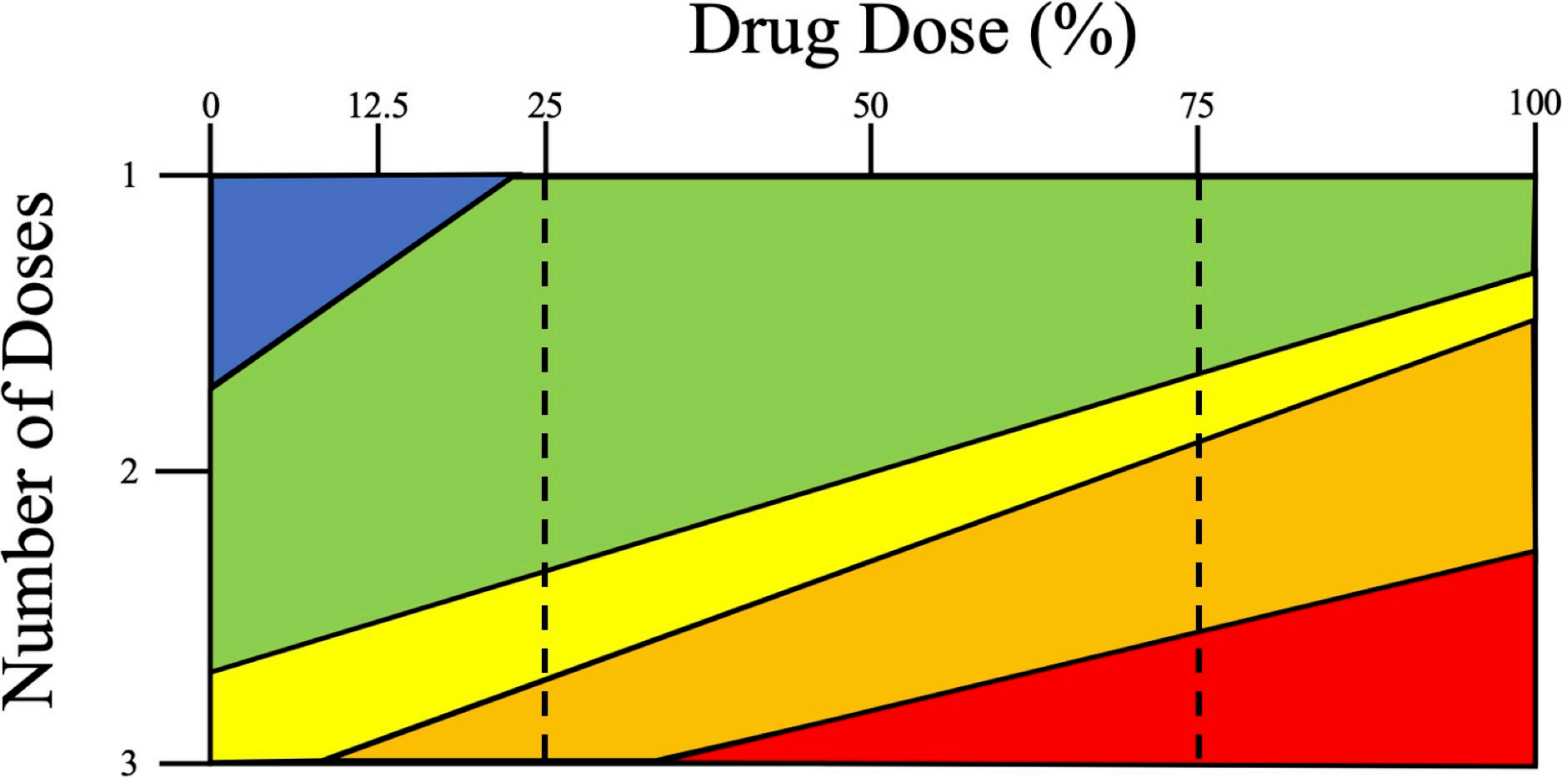
Drug dose concentration and number of doses vs parasite survival. Examining recovery times after treatment allows for comparison between drug concentrations and adherence schedules tested. The pharmacologically relevant dose of DHA is 700 nM (100%) and is typically administered once a day for 3 days. Colors represent the time between drug treatment and re-establishment of visible growth in culture. Red = over 3 weeks; orange = over 2 weeks; yellow = over 1 week; green = less than one week; blue = less than one week and CamWT outcompetes CamWT^C580Y^ shortly after recrudescence. The number of doses impacts parasite killing more than the concentration; however, best results are seen with full adherence and higher concentrations of drugs. Experimental data was used in order to create summary figure.

Testing different combinations of drug concentrations and doses (see Fig 1B and Figs 4–6 for results) allows us to summarize the relative fitness of the genetically matched pair of ARTS:ARTR strains (CamWT:CamWT^C580Y^) under these different drug exposure conditions (Fig 7). Interestingly, changes in the time of mixed culture recovery are more closely associated with the number of doses than with the [DHA]. The clearest example of this is the set of data for 25% (175 nM) dosing (left dashed vertical line, Fig 7). Mixed cultures bolus dosed with 25% DHA took less than one week (green), vs over one week (yellow), or vs over two weeks (orange) to reemerge upon one, two, and three doses, respectively. However, this does not discount the concentration of drug as an additional important variable, as the greatest killing was seen when combining a full adherence schedule with clinically relevant [DHA] (red). Extrapolating all data collected permits us to predict effects for other drug concentrations (e.g. 75%, Fig 7 right dashed line). We found that parasites that are bolus dosed three times with concentrations at or above 350 nM DHA (≥ 50% of the pharmacologically relevant dose) showed at least three weeks of lag time between the last drug exposure and visible parasitemia (Fig 7).

**Fig 8:**
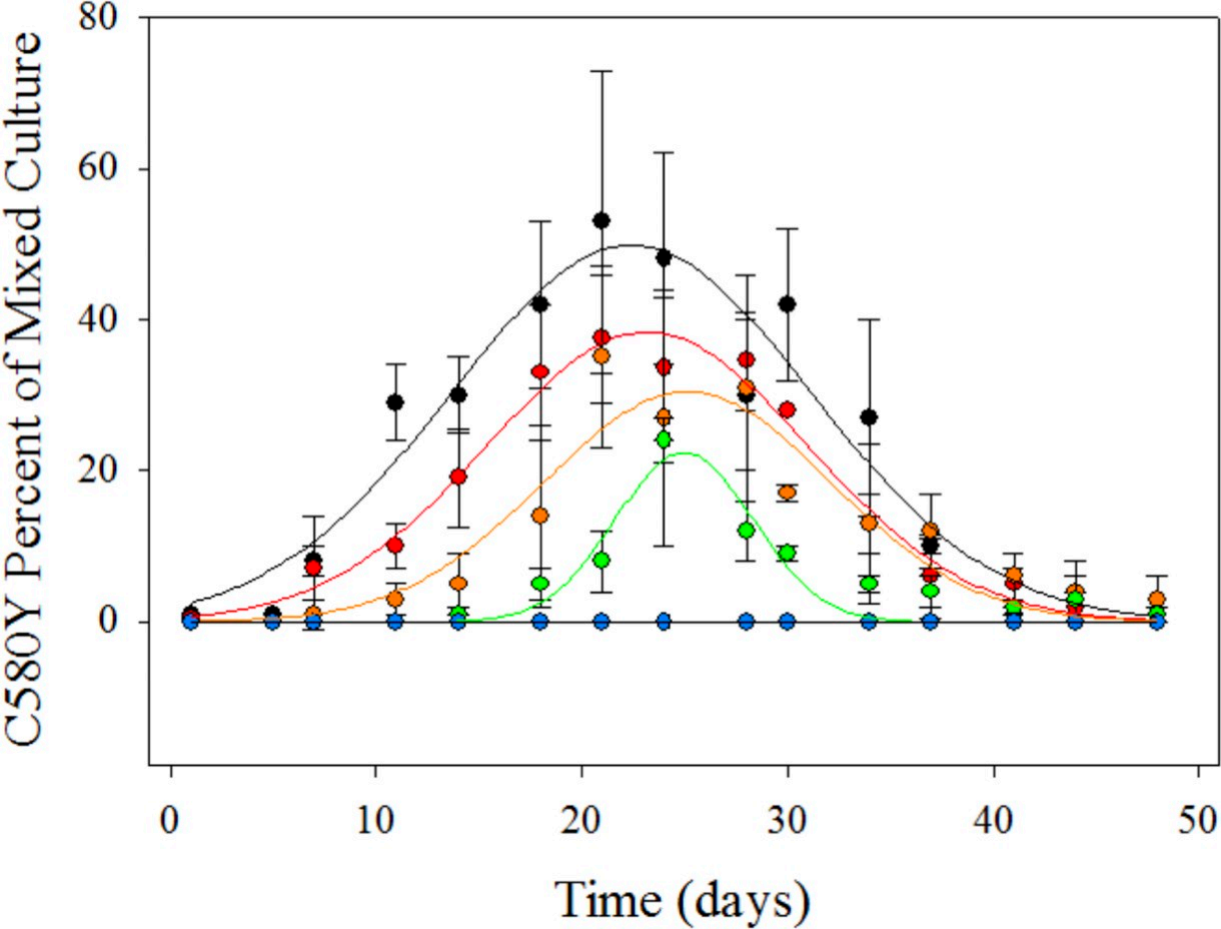
Mixed culture starting ratio titration outgrowth following DHA bolus dose. CamWT and CamWT^C580Y^ were mixed at varying starting ratios in complete media and dosed with DHA once at 700 nM for 6 h. Starting ratios (CamWT^C580Y^:CamWT) analyzed were 1:99 (black), 1:174 (red), 1:249 (orange), 1:499 (green), 1:749 (blue), and 1:999 (yellow). Measurable outgrowth of CamWT^C580Y^ was identified for 1:99, 1:174, 1:249 and 1:499 starting ratios. However, unlike the 50:50 starting ratio experimental set-up, there was not a long-term fitness advantage identified for CamWT^C580Y^ under these conditions. Error bars represent the SEM of at least three independent trials.

In addition to 50:50 ratio mixed cultures, we also examined various different starting ratios of parasites. We endeavored to examine mixtures of ARTS vs ARTR parasites that might be found in a patient. A 50:50 ratio represents a simplistic “macroscopic” view of the GMS, since many regions experiencing ACT failure harbor close to this ratio of ARTS:ARTR parasites across the region [2]. However, it is less likely that an individual patient would be infected with such a ratio. We therefore examined a range of ratios where CamWT^C580Y^ parasites were seeded at lower concentration than CamWT. Fig 8 shows data for different starting ratios of parasites that were treated with a single dose of 700 nM DHA for 6 h. The ratios examined were 1:99, 1:174, 1:249. 1:499, 1:749, and 1:999 (CamWT^C580Y^:CamWT). Ratios as low as 1:499 still show a measurable outgrowth of CamWT^C580Y^ parasites over CamWT parasites for ~ 3 weeks. However, the fitness advantage under such conditions is not able to overcome the differences in starting ratios and the CamWT strain eventually outcompetes CamWT^C580Y^ due to its faster growth rate relative to the intrinsically fitness-compromised ARTR strain (Fig 2). The 1:99 ratio (black symbols) was the only condition where CamWT^C580Y^ parasites became more than 50% of the culture within the monitored time window (Fig 8).

## Discussion

Results in this paper can be summarized as follows:

1. In the absence of drug, CamWT is more fit than CamWT^C580Y^ within red cell culture, suggesting that a 580Y substitution within PfK13 confers a fitness cost to malarial parasites.
2. Drug pressure creates an environment that favors proliferation of mutant PfK13-mediated ARTR parasites over isogenic ARTS.
3. Subtherapeutic concentrations of DHA favor the spread and proliferation of ARTR parasites in our tissue-culture competition experiments with the relative degree related to both drug concentration and number of doses.
4. These results allow us to predict behavior at untested conditions that may be of use in predicting the future spread of ARTR.

Changes in parasite fitness are often a consequence of drug resistance conferring mutations. This phenomenon has been observed previously in *P*. *falciparum* and suggests that mutations that confer increased drug resistance often result in decreased fitness (reduced growth rate) in the absence of drug [41–47]. However, to date, there is conflicting evidence regarding the impact of *pfk13* mutations on overall parasite fitness [2, 12, 21, 41–45]. Three studies suggest that parasites harboring *pfk13* mutations are less fit than their WT counterparts [41, 43, 45], but one of these suggests the fitness costs of a C580Y propeller domain substitution may be negligible in certain genetic backgrounds [41].

Other studies have suggested that a fitness disadvantage is intrinsic to some ARTR parasite lines, however the precise impact of *pfk13* mutations in conferring these phenotypes is difficult to ascertain [2, 12, 21, 42, 44] due to additional differences between the ARTS and ARTR parasites being compared. In another study it was reported that an ARTR line was the most fit out of all ARTS and ARTR lines tested [44]. Tirrell *et al*. [44], culture adapted 7 isolates from the Thailand-Myanmar border with varying genetic backgrounds, clearance times after ACT treatment, and K13 polymorphisms and then ranked the relative fitness of the laboratory adapted lines (in the absence of drug) [44]. Including a control drug sensitive line (NF54), the most fit line was observed to be an ARTR parasite with an elongated parasite clearance half-life that did not show *pfk13* mutation. This was an interesting observation xsince over the past decade parasites harboring wild type *pfk13* appear to have been selected against as evidenced by their decreasing frequency in SEA [44]. Surprisingly, the most fit line and the least fit line were both wild type *pfk13* yet ARTR parasites showed elongated parasite clearance half-times [44]. The impact of *pfk13* mutations on parasite fitness therefore cannot be definitively determined from this analysis.

In our analysis we find that there is a measurable fitness cost associated with expression of C580Y mutant PfK13 protein. Fig 2 clearly illustrates this point, as CamWT outcompetes CamWT^C580Y^ in a mixed culture in the absence of drug pressure within 2 weeks. Our data suggests that in the absence of drug pressure in the field, and assuming the ARTR phenotype is caused only by *pfk13* mutation, it is likely that an otherwise genetically similar ARTS strains would outcompete the ARTR strain. In addition, although one laboratory has previously examined the impact of drug pressure on ARTR strain fitness [42], to our knowledge, the present study is the first to examine the impact of multiple DHA drug doses as well as [DHA] on the relative fitness of ARTS vs mutant PfK13-mediated ARTR parasites. In our hands, even low concentrations of DHA can impart at least a temporary fitness advantage to CamWT^C580Y^. However, importantly, the concentration of drug and the number of doses given impacts the time to outgrowth in culture as well as re-emergence (if any) of ARTS CamWT within the culture.

It should not be surprising that ARTR strains outcompete ARTS in the presence of drug. What is surprising is the altered degree to which ARTR parasites thrive compared to their isogenic ARTS parent after variable drug treatment. A single 6 h bolus dose of DHA, at concentrations greater than 12.5% of the pharmacologically relevant dose, creates enough of a fitness advantage for ARTR parasites to selectively proliferate for the length of our observation period (see Figs 5B, 6B and 7B). This may help to explain how a single C580Y lineage from western Cambodia spread throughout the rest of the GMS, where ART-based drug monotherapy had been prevalent [5, 54–56]. Survey of poor-quality medicines from around the world shows that substandard or falsified medicines are not isolated to a specific region [26–40]. Evidence of poor-quality medicines has been documented over the last decade in Africa [30, 34, 37] and Southeast Asia [27, 33, 36] the two areas of the world most plagued by malaria. While the C580Y substitution may not provide as robust a level of resistance to ARTs relative to some other propeller mutations [20], it may harbor the most advantageous combination of minimal fitness disadvantage and ARTR to allow for its nearly ubiquitous spread in the GMS.

However, at sufficiently low concentrations of drug, there is not enough selective pressure on the mixed culture for the sustained outgrowth of the ARTR parasites. At concentrations equal to or below 12.5% of the pharmacologically relevant dose, a small initial fitness advantage is seen for CamWT^C580Y^ but the drug pressure applied to the mixed culture is not enough to overcome the intrinsic fitness gap between the two strains (Fig 4). This suggests that removal of drug pressure from the clinic could allow for the re-emergence of ARTS strains, which has been observed in the field before with other antimalarial drugs such as chloroquine [57–60]. Unfortunately, since there are currently no effective alternatives to ACTs, removal of drug pressure is not a viable option to reduce the spread of ARTR. Interestingly, a second (or third) dose at these lower concentrations (12.5% and 6.25%) provided a fitness advantage for CamWT^C580Y^ that lasted the length of the entire experiment, suggesting that multiple dosing regimens impart a higher selective pressure relative to single dosing.

Other forms of drug resistance, including to ACT partner drugs (particularly PPQ), are also evolving in the GMS. The dominant parasite lineage harboring a C580Y PfK13 substitution that has been spreading throughout the GMS since 2017 also shows elevated PPQ tolerance [54, 55]. Multiple genetic markers have been associated with PPQ resistance making analysis of the situation complicated [61–66]. Amplification of *plasmepsin2* and *plasmepsin3* and mutation of *pfcrt* have all been associated with increased resistance to PPQ, [62–66]. Nonetheless, increased frequency of PPQ failures in association with ARTR parasites suggests subtherapeutic dosing of ACT drug should be examined in strains that model both resistances as well.

We note that only asexual stages of parasite development were examined in our competition assays. These are arguably the most important stages of parasite development from a clinical perspective, as these are the stages where symptoms are manifested in the human host, however, such competition assays obviously do not fully capture all aspects of parasite fitness that may be relevant in the wild. Regardless, in addition to observing a drug concentration dependent fitness advantage for CamWT^C580Y^ we find that multiple DHA doses provide greater parasite kill relative to increased concentration of drug. Parasite killing was calculated to be 1–2 logs with a single dose depending on the drug concentration, with a 4–5 log drop in parasitemia observed after three bolus doses of DHA whereas increasing the concentration of DHA 16-fold (from 6.25% [43.8 nM] to 100% [700 nM]) only varied parasite killing by one log, suggesting that the number of doses is more critical to parasite elimination than the drug concentration, and illustrating circumstances where varying treatment might promote increased spread of ARTR parasites. Fig 7 models the impact of adherence schedule vs. drug dose.

An important possibility that cannot be fully modelled in this study is enigmatic drug induced parasite “dormancy”. It has been suggested that after ART treatment, parasites can enter into an incompletely defined “dormant” state where parasite development stalls at the ring stage of red cell development [67, 68]. The ring stage appears particularly important for resistance, as this is the stage measured in the RSA and the stage at which resistance is most clearly manifested. Our experimental approach examines asynchronous parasites, with number of rings not precisely quantified. Regardless, a higher proportion of ARTR parasites are theorized to recover more quickly from such a dormant state, which may help to explain why more ARTR parasites survive drug exposure. It has been reported that laboratory pressured ARTR parasites recovered from drug pressure and resumed growth faster than sensitive parental lines after proportional drug treatments [69] and that protein turnover for a R539T PfK13 edited line resumed faster after drug exposure than did isogenic wild type PfK13-expressing parasites [70].

Some evidence for such altered states of parasite growth for ARTS vs ARTR parasites is shown in Fig 8. That is, outgrowth of ARTR parasites (Figs 4–6) can be explained in one of two ways. Either the initial kill of parasites post drug treatment is orders of magnitude different for CamWT vs CamWT^C580Y^ (which seems unlikely), or the growth rate of CamWT^C580Y^ is faster (for some period of time) than CamWT post drug treatment. The data in Fig 8 suggests that for a certain amount of time the growth rate for CamWT^C580Y^ is faster. If there was a much larger initial kill for CamWT compared to CamWT^C580Y^, the peaks seen in Fig 8 would be seen immediately after drug treatment, and CamWT would slowly outcompete CamWT^C580Y^ over time. Instead, we do not see a significant change in the starting ratio for a number of days or weeks, and for a period of time, CamWT^C580Y^ grows faster than CamWT (represented by the slow rise of the %ARTR culture seen in Fig 8), until ~Day 25 when CamWT starts to outcompete CamWT^C580Y^, which indicates that CamWT’s growth rate is now faster post Day 25 (represented by the decrease in peak height in Fig 8). That is, for a period of time, CamWT^C580Y^ grows faster than CamWT until ~Day 25 when CamWT then outcompetes CamWT^C580Y^, which indicates that growth of CamWT is now faster (represented by the decrease in peak height in Fig 8). Additional modelling of mixed cultures at variable ratio, drug dose, and drug dosing schedule, should prove informative. Additional work is needed to adequately quantify variable dormancy periods after variable drug treatment.

Under some conditions subtherapeutic doses of ART-based drug favor the proliferation of ARTR parasites in our tissue-culture competition experiments. Even at lower concentrations where CamWT parasites eventually outcompeted CamWT^C580Y^ (expressed as % of the culture), initially there were larger numbers of surviving ARTR parasites and an initially sustained fitness advantage of CamWT^C580Y^ parasites. Transmission modelling for resistant parasites at these concentrations would be dependent upon the length of time between administration of subtherapeutic concentrations of drug and actual transmission. At higher drug concentrations, there is a prolonged outgrowth of CamWT^C580Y^ parasites post DHA treatment but initially at much lower numbers.

ACTs are currently the last line of defense we have to effectively treat all malaria in the clinic. It is critical to halt the spread of ARTR and ACT partner drug resistances if we are to prevent this epidemic from spreading further into SEA or into Africa. This study provides data relevant to how adequate treatment may be important for reducing the burden of ARTR. Special attention must be given to providing full regimens of ACTs as prescribed by the WHO as well as identifying and removing substandard and falsified medicines in markets where treatment is most critical. These findings suggest that substandard antimalarials may foster the spread of resistant pathogens in patient populations; medicine quality, therefore, is deserving of policymaker consideration as decisions are made about where to direct investments to improve public health.

## Supporting information

S1 File Raw data plotted in Figs 4–6 and 8 of the manuscript. (XLSX)
